# Editorial: Insights in Evolutionary and Genomic Microbiology: 2021

**DOI:** 10.3389/fmicb.2022.915593

**Published:** 2022-05-18

**Authors:** Daniel Yero, Baolei Jia, Feng Gao

**Affiliations:** ^1^Institut de Biotecnologia i de Biomedicina (IBB), Universitat Autònoma de Barcelona (UAB), Barcelona, Spain; ^2^Departament de Genètica i de Microbiologia, Universitat Autònoma de Barcelona (UAB), Barcelona, Spain; ^3^Department of Life Science, Chung-Ang University, Seoul, South Korea; ^4^Department of Physics, School of Science, Tianjin University, Tianjin, China; ^5^Frontiers Science Center for Synthetic Biology, Key Laboratory of Systems Bioengineering (Ministry of Education), Tianjin University, Tianjin, China

**Keywords:** next generation sequencing, long-read sequencing, comparative genomics, adaptive evolution, pan-genome

Recent advances in DNA sequencing and bioinformatics have shaped and accelerated recent advances in all fields of microbiology (Gao, 2019; Kobras et al., 2021). Next-generation sequencing (NGS) together with large-scale genomic comparisons allows, among others, identifying the extent of genetic variability among related isolates and strains, and a more comprehensive analysis of the evolution of adaptation, antibiotic resistance, and pathogenicity factors in clinically relevant pathogens. In line with this, one study by Whaley et al. characterizes the capsule polysaccharide synthesis genes in 1,514 isolates of *Neisseria meningitidis* by whole-genome sequencing (WGS). This study shows that among meningococcal carriage isolates collected from student populations at three US universities, several mutations allow the bacteria to switch between an encapsulated and non-encapsulated state. The importance of this study lies in the fact that variations in capsule polysaccharide synthesis may potentially result in polysaccharide-based meningococcal vaccine escape. A second study by Manoharan-Basil et al. provides a current comprehensive overview of the role that horizontal gene transfer (HGT) plays in the evolution of antibiotic resistance in *Neisseria gonorrhoeae* by using WGS data comprising 20,047 isolates. This study focuses on the global spread and emergence of fluoroquinolone resistance in gonococci and highlights the significant role that HGT plays in transferring resistance from commensal *Neisseria* species to pathogenic *Neisseria*.

In a multinational and multicenter study by Topaz et al., authors present the phylogenetic structure and comparative genomics of 410 invasive *Haemophilus influenzae* serotype a (Hia) isolates. In addition to identifying genetic differences in virulence and antimicrobial resistance genes and describing the phylogenetic structure of Hia, a genome-wide association study was conducted in order to detect associations with clinical and epidemiological traits. They show that very little of the phenotypic variation was due to the genetic variability present in the studied population, at least for the variables analyzed. Another study by Yuan et al. presents a pan-genome analysis of *Laribacter hongkongensis*, a potential emerging pathogen, which leads to the study of the distribution of virulence and antimicrobial resistance genes among strains of different sources. Authors assess the influence of the source and lineage of strains on pathogenicity risk, suggesting that strains isolated from frogs may have a higher potential to become human pathogens.

The increase in genomic information for some bacterial species has allowed molecular taxonomy and environmental adaptation to be studied in depth. For instance, the complete genome of *Acidiferrobacter thiooxydans* is presented for the first time in a work by Ma et al. (only a partial genome at NCBI at the time of this publication). Analysis of this sequence and comparative genomic studies reveal a unique phylogenetic position and a genomic plasticity that contributes to the environmental adaptation of these bacteria in extremely acidic niches with high metal concentrations. In another study by Kong et al., the complete genome of a new strain of *Rahnella victoriana* also reveals new insights into its molecular and genetic mechanism for promoting plant growth. Among the beneficial features, the new genome carries genetic information for indole-3-acetic acid production, volatile organic compound biosynthesis, nitrogen fixation, phosphate solubilization, siderophores, acetoin, 1-aminocyclopropane-1-carboxylate deaminase, and gamma-aminobutyric acid production.

In this Research Topic some studies use long-read WGS, a third-generation sequencing technology, to generate genomic data. Long-read techniques offer advantages over short-read sequencing because they are well suited to the detection of larger sequence changes such as structural variants and phased variations, and they have the ability to span repetitive regions (Amarasinghe et al., 2020). A study by Thibau et al. shows a conserved genome sequence among eight *Bartonella henselae* isolates and identifies a variable genomic island by using PacBio single molecule real-time (SMRT) sequencing. In a second study by Abdel-Glil et al., genome sequencing using the PacBio SMRT method was used to establish phylogenetic relatedness and the genome structure of *Yersinia ruckeri* isolates. Comparative genomics shows that *Y. ruckeri* has unique genomic regions probably related to the pathogenesis of enteric redmouth disease in fish.

Recent advances in the sequencing and analysis of bacterial genomes have also shed light on the adaptive evolution mechanisms. One study by Kurokawa et al. presents works on experimental evolution in *Escherichia coli* strains with different genome sizes with the aim to determine a connection between genome reduction with adaptation to environmental gradients and the contribution of mutations to bacterial fitness. The results reveal a quantitative relationship among genome reduction, adaptation, and niche expansion. A second study by Zhao et al. focuses on genomic island evolution as important adaptive traits in pathogenic or environmental bacteria. By using comparative genomics, a new composite island was identified and characterized in *Shewanella putrefaciens* carrying a functional HipAB toxin–antitoxin system. Finally, another study by Garzón et al. explored a novel *in silico* approach based on bacterial genomic data to propose a minimal translational machinery needed for protein synthesis. This information could contribute to the design of synthetic cells for biotechnological purposes.

## Author Contributions

All authors listed have made a substantial, direct, and intellectual contribution to the work and approved it for publication.

## Funding

FG is supported by the National Key Research and Development Program of China (Grant Number 2018YFA0903700) and the National Natural Science Foundation of China (Grant Numbers 21621004 and 31571358).

## Conflict of Interest

The authors declare that the research was conducted in the absence of any commercial or financial relationships that could be construed as a potential conflict of interest.

## Publisher's Note

All claims expressed in this article are solely those of the authors and do not necessarily represent those of their affiliated organizations, or those of the publisher, the editors and the reviewers. Any product that may be evaluated in this article, or claim that may be made by its manufacturer, is not guaranteed or endorsed by the publisher.
